# Design and synthesis of benzothiazole-based SLC-0111 analogues as new inhibitors for the cancer-associated carbonic anhydrase isoforms IX and XII

**DOI:** 10.1080/14756366.2022.2124409

**Published:** 2022-09-22

**Authors:** Tarfah Al-Warhi, Mostafa M. Elbadawi, Alessandro Bonardi, Alessio Nocentini, Ahmed A. Al-Karmalawy, Nada Aljaeed, Ohoud J. Alotaibi, Hatem A. Abdel-Aziz, Claudiu T. Supuran, Wagdy M. Eldehna

**Affiliations:** a Department of Chemistry, College of Science, Princess Nourah bint Abdulrahman University, Riyadh, Saudi Arabia; b Department of Pharmaceutical Chemistry, Faculty of Pharmacy, Kafrelsheikh University, Kafr el-sheikh, Egypt; c Department of NE.UROFARBA, Section of Pharmaceutical and Nutraceutical Sciences, University of Florence, Polo Scientifico, Firenze, Italy; d Department of Pharmaceutical Medicinal Chemistry, Faculty of Pharmacy, Horus University-Egypt, Egypt; e Department of Applied Organic Chemistry, National Research Center, Dokki, Egypt

**Keywords:** Metalloenzymes, sulphonamides, anticancer agents, hypoxic tumours, ureido derivatives

## Abstract

In this work, different series of benzothiazole-based sulphonamides **8a-c, 10, 12, 16a-b** and carboxylic acids **14a-c** were developed as novel SLC-0111 analogues with the goal of generating potent carbonic anhydrase (CA) inhibitors. The adopted strategy involved replacing the 4-fluorophenyl tail in SLC-0111 with a benzothiazole motif that attached to the ureido linker to produce compounds **8c** and its regioisomers **8a-b**. In addition, the ureido spacer was elongated by methylene or ethylene groups to afford the counterparts **10** and **12**. In turn, the primary sulfamoyl zinc binding group (ZBG) was either substituted or replaced by carboxylic acid functionality in order to provide the secondary sulphonamide-based SLC-0111 analogues **16a-b**, and the carboxylic acid derivatives **14a-c**, respectively. All compounds (**8a-c, 10, 12, 14a-c** and **16a-b**) were tested for their ability to inhibit CA isoforms CA I, II, IX and XII. Additionally, the *in vitro* anticancer properties of the developed CAIs were evaluated.

## Introduction

Carbonic anhydrases (CAs, EC 4.2.1.1) are family of ubiquitous zinc-metalloenzymes present in the whole organisms^1^. These enzymes catalyse the essential conversion of carbon dioxide and water to bicarbonate and proton in a crucial process accountable for diverse cellular activities such as electrolyte secretion, bone resorption, maintenance of acid-base balance, gluconeogenesis, CO_2_ and pH homeostasis, calcification and tumorgenicity^2–4^. The human CAs (*h*CAs) are relevant to α-CAs isozymes and sub-categorized into fifteen isoforms displaying distinct cellular distribution, levels of expression, kinetics and molecular features^5^^,^^6^. Of special interest, the catalytic activity of CAs I-IV, VA, VB, VI, VII, IX, XII-XIV isoforms is due to the presence of three histidine residues in the active site in coordination with zinc^7^. Furthermore, *h*CAs are classified upon cellular distribution into cytosolic (*h*CAs I, II, III, VII and XIII), trans membrane (*h*CAs IV, IX, XII, and XIV), mitochondrial (*h*CAs VA and VB), and *h*CA VI is secreted in milk and saliva^8^^,^^9^. The abnormal levels of these enzymes have been associated with many diseases; thus inhibitors of the CAs have potential applications in the treatment of glaucoma, edoema, obesity and mental problems^1^^,^^7^^,^^9^^,^^10^.

Interestingly, the trans-membranal *h*CA IX and XII have shown diverse peculiarities over the other isoforms. *h*CA IX/XII isozymes have shown elevated expression in tumour cells and are associated to hypoxic solid tumours inducing tumour growth and metastasis^11^^,^^12^. Accordingly, the extremely desired selective inhibition of cancer-associated *h*CA IX/XII has been described as cutting-edge approach for the discovery of new small molecules for cancer treatment^13–19^. As a consequence, several approaches have been employed to develop selective inhibitors for *h*CA IX/XII. Strikingly, tail approach stood out as the most successful and effective tool to improve the potency and selectivity of sulphonamide-based carbonic anhydrase inhibitors (CAIs)^20^. In this context, tail approach has been devoted to generate selective sulphonamide CAIs which involves grafting of different molecular motifs (tails) to the aromatic/heterocyclic ring bearing a zinc binding group (ZBG) through a flexible spacer^21^^,^^22^.

**Figure 1.**
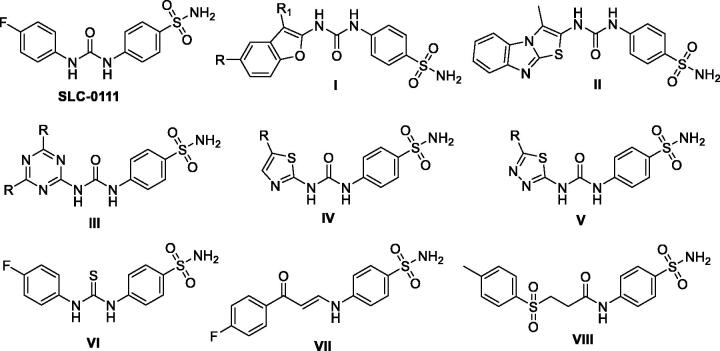
SLC-0111 chemical structure, and some of the previously reported SLC-0111 analogues.

The ureido benzenesulfonamide SLC-0111 (Figure 1) has been developed utilising the tail approach as the first-in-class *h*CA IX inhibitor which, for the management of metastatic hypoxic solid tumours, is currently being investigated within phase I/II clinical trials^3^^,^^23^^,^^24^. To date, numerous SLC-0111 analogues have been described with the prime aim to enhance potency and selectivity towards *h*CA IX exploiting bioisosteric replacement approach *via* replacement of the SLC-0111aryl tail by various sets of chemical scaffolds (Figure 1) like benzofuran **I**^23^, thiazolo[3,2-*a*]benzimidazole **II**^25^, triazine **III**^26^, thiazole **IV**, and thiadiazole **V**^27^. In addition, the replacement of the ureido linker in SLC-0111 with another spacers (Figure 1) for instance, thioureido **VI**^14^, enaminone **VII**^28^ and sulfonylpropanamide **VIII**^3^ was adopted for developing novel SLC-0111 analogous. Remarkably, these strategies resulted in an enhancement of tumour-associated *h*CA IX inhibitory action.

**Figure 2.**
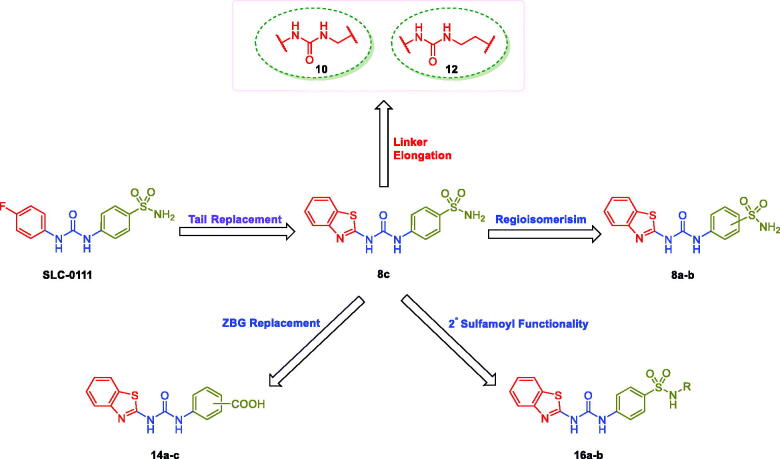
Design of the target benzothiazole-based SLC-0111 analogues as CAIs.

The aforesaid results motivated our research interest to adopt the tail approach for the development of new SLC-0111 analogues featured with potent and selective *h*CA IX/XII inhibitory influence. In the herein study, the benzothiazole motif has been appended to the ureido linker rather than the phenyl tail in SLC-0111 in order to provide the target inhibitor **8c** (Figure 2). Thereafter, the regioisomers **8a-b** were designed through shifting of sulfamoyl functionality in **8c** to *ortho* and *para* positions, respectively (Figure 2). In addition to this, the ureido spacer in the SLC-0111 analogue **8c** was lengthened by either methylene or ethylene groups, which resulted in the counterparts **10** and **12**, respectively. Moreover, the functionality of the primary ZBG was either replaced by carboxylic acid or it was substituted in order to produce the carboxylic acid derivatives **14a-c** and the secondary sulphonamide-based SLC-0111 analogues **16a-b**, respectively.

The target benzothiazole-based SLC-0111 analogues **8a-c**, **10**, **12**, **14a-c** and **16a-b** were developed and screened for their inhibitory impact towards CA I, II, IX and XII isoforms. Furthermore, the designed CAIs were assessed for their potential *in vitro* anticancer effects.

## Experimental

### Chemistry

#### General

Uncorrected melting points were measured using a Stuart melting point device. In addition, the Schimadzu FT-IR 8400S spectrophotometer was used to record the IR spectra, whereas the Bruker spectrophotometer (400 MHz) was used to record the NMR spectra. ^13^C NMR spectra were run at 100 MHz in deuterated dimethylsulphoxide (DMSO-*d_6_*). All coupling constant (*J*) values are reported in hertz. Both ethyl 1,3-benzothiazole-2-carboxylate **3** and benzo[*d*]thiazole-2-carbohydrazide **4** were prepared as previously reported^29^.

#### Ethyl benzo[d]thiazole-2-carboxylate 3

White crystals, m.p. = 69–72 °C (reported m.p. = 68–69 °C)^29^, yield = 77%.

#### Benzo[d]thiazole-2-carbohydrazide 4

White crystals, m.p. = 174–175 °C (reported m.p. = 173–174 °C)^29^, yield = 84%.

#### General procedures for the preparation of target benzothiazole-derived sulphonamides 8a-c, 10, 12, 16a-b and the carboxylic acids 14a-c

A mixture of benzo[*d*]thiazole-2-carbohydrazide **4** (0.58 g, 3 mmol) and sodium nitrite (0.41 g, 6 mmol) in glacial acetic acid was stirred in an ice bath for 2 h. Azide **4** was produced by air-drying the generated solid, washing it with water (3 × 4 mL), and collecting it using filtration. Azide **4** was then heated for 30 min at reflux in dry xylene. To the prepared xylene solution, the appropriate amine derivative (aminobenzenesulfonamides **7a-c**, 4-(aminomethyl)benzenesulfonamide **9**, 4–(2-aminoethyl)benzenesulfonamide **11**, aminobenzoic acids **13a-c**, and secondary sulphonamides **15a-b**) was added. After being refluxed for four hours, the reaction mixture was allowed to settle down to room temperature. The desired benzothiazole sulphonamides and carboxylic acids were obtained by filtering, washing the formed precipitate with methylene chloride (3 × 3 mL), drying it, and recrystallizing it from isopropyl alcohol.

Both spectral (NMR and IR) and elemental analysis for the newly prepared sulphonamides (**8a-c**, **10**, **12**, and **16a-b**) and carboxylic acids (**14a-c**) were described in the Supplementary Materials.

### Biological evaluation

The procedures that were used for the conducted biological tests were carried out in the same manner that was stated earlier; stopped-flow CA^30–34^, NCI-single dose^35–37^ and MTT cytotoxicity^38–41^ assays, and they were detailed in the Supplementary Materials.

## Results and discussion

### Chemistry

**Scheme 1.**
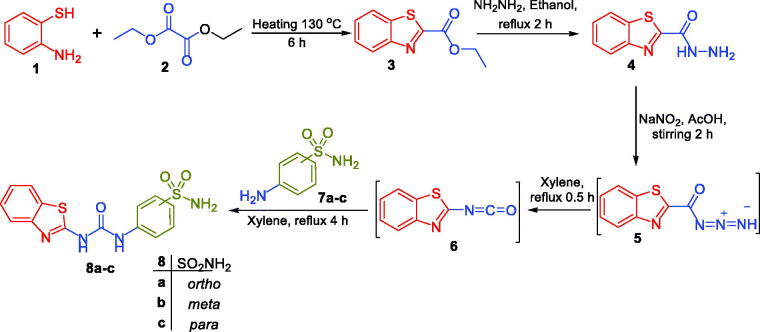
Synthesis of target benzothiazole-derived sulphonamides **8a-c**.

**Scheme 2.**
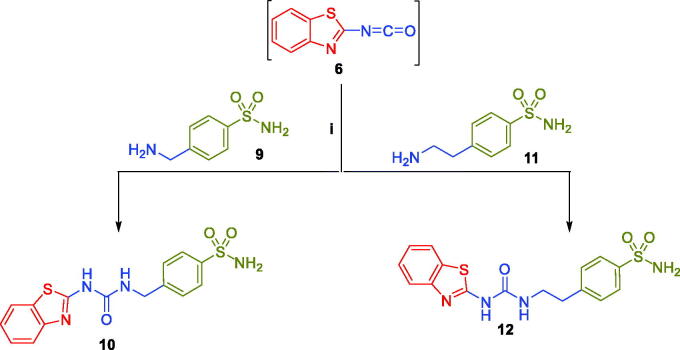
Synthesis of target benzothiazole-derived sulphonamides **10** and **12**.

**Scheme 3.**
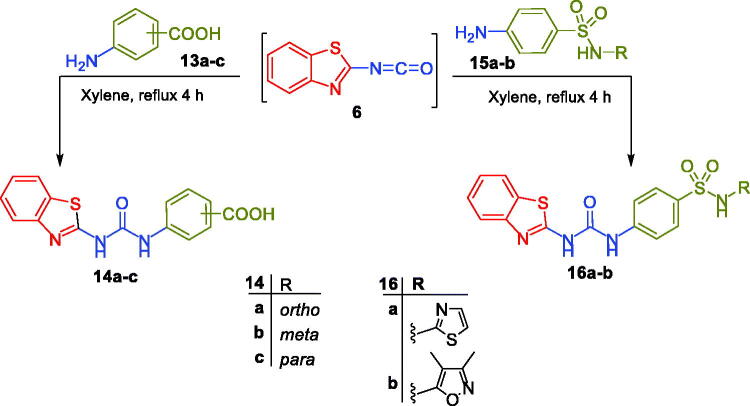
Synthesis of target benzothiazole-derived carboxylic acids **14a-c** and sulphonamides **16a-b**.

Schemes **1**, **2**, and **3** illustrate the synthetic routes that were used in order to prepare the target benzothiazole-derived sulphonamides **8a-c, 10, 12**, and **16a-b**, as well as the carboxylic acids **14a-c**.

Synthesis was started by cyclisation of 2-aminothiophenol **1** with diethyl oxalate **2** to afford ethyl benzo[*d*]thiazole-2-carboxylate **3.** Hydrolysis of ester **2**, *via* refluxing with hydrazine hydrate in ethanol, afforded benzo[*d*]thiazole-2-carbohydrazide **4** in 84% yield. Thereafter, stirring of intermediate **4** with sodium nitrite in glacial acetic acid at 0 °C produced the corresponding azide derivative **5**. In the last step, preparation of target benzothiazole-derived sulphonamides **8a-c** was performed *via* the addition of the appropriate aminobenzenesulfonamide derivative **7a-c** to a pre‐heated azide **4** solution in xylene (Scheme 1).

Furthermore, 2-isocyanatobenzothiazole intermediate **6** was reacted with 4-(aminomethyl)benzenesulfonamide **9** and 4–(2-aminoethyl)benzenesulfonamide **11** in refluxing xylene to yield target benzothiazole-derived sulphonamides **10 and 12**, respectively (Scheme 2).

In the last Scheme, benzothiazole-based carboxylic acids **14a-c** and sulphonamides **16a-b** were obtained through a nucleophilic addition reaction of 2-isocyanatobenzothiazole intermediate **6** with aminobenzoic acids **13a-c** and secondary sulphonamides **15a-b**, respectively (Scheme 3).

### Biological evaluation

#### Carbonic anhydrases inhibition

The herein synthesised benzothiazole-derived sulphonamides **8a-c**, **10**, **12**, **16a-b** and the carboxylic acids **14a-c** were assessed for their inhibitory action against the widespread cytosolic *h*CA I and II, and cancer-related IX and XII isoforms employing a stopped flow CO_2_ hydrase assay and the CAI acetazolamide (**AAZ**) was adopted as a control^30^. The provided inhibition constants (K_I_) manifested in Table 1 can be exploited to delineate the structure activity relationships (SARs).

**Table 1.** Inhibition constants for benzothiazole-based derivatives (**8a-c, 10, 12, 14a-c** and **16a-b**) and the standard sulphonamide inhibitor acetazolamide (**AAZ**) towards *h*CA I, II, IX and XII, determined with a stopped-flow CO_2_ hydrase assay.

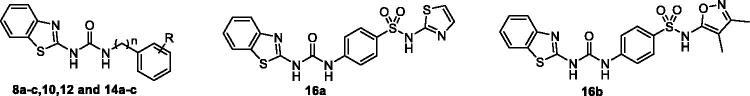						
Cmpd	*n*	R	K_I_ (nM)^a^			
			hCA I	hCA II	hCA IX	hCA XII
**8a**	0	*o*-SO_2_NH_2_	12590	785.2	65.3	41.2
**8b**	0	*m*-SO_2_NH_2_	4040	652.7	48.9	57.5
**8c**	0	*p*-SO_2_NH_2_	361.7	54.1	31.5	29.3
**10**	1	*p*-SO_2_NH_2_	945.9	204.3	58.8	51.2
**12**	2	*p*-SO_2_NH_2_	61.5	28.5	16.4	34.7
**14a**	0	*o*-COOH	68090	75940	44620	39860
**14b**	0	*m*-COOH	82750	94130	16280	9140
**14c**	0	*p*-COOH	11820	9470	2410	8540
**16a**	–	–	>100000	>100000	56090	32500
**16b**	–	–	>100000	>100000	>100000	>100000
**AAZ**			250.0	12.5	25.0	5.7
**SLC-0111**			5080.0	960.0	45.0	4.5

^a^Mean from three different assays (errors were in the range of ± 5–10% of the reported values).

The herein tested benzothiazole-based sulphonamides **8a-c**, **10**, **12**, **16a-b** and the carboxylic acids **14a-c** displayed diverse off-target *h*CA I inhibition profile spanning from nanomolar to high micromolar inhibitory constants (K_I_s = 61.5 nM to > 100 µM), Table 1. In the regard of benzothiazole-bearing sulphonamides with ureido linker **8a-c**, they exerted low to moderate inhibitory activities towards the dominant *h*CA I with K_I_s ranged from 361.7 nM to 12.59 µM. Noteworthy, the switching of sulphonamide anchoring moiety from *ortho*- and *meta* positions (**8a-b**; K_I_s = 12.59 and 4.04 µM, respectively) to *para* position potentially elevated the inhibition constant to the nanomolar level (**8c**; K_I_ = 361.7 nM). Notably, the elongation of the ureido spacer in the *p*-benzenesulfonamide counterpart **8c** (K_I_ = 361.7 nM) by one carbon sharply reduced *h*CA I inhibitory potency (**10**; K_I_ = 945.9 nM), whereas its elongation by two carbons led to sensible enhancement in *h*CA I inhibitory impact providing the most potent *h*CA I inhibitor within the current study (**12**; K_I_ = 61.5 nM). In contrast, the bioisosteric replacement of the sulphonamide zinc binding functionality in **8a-c** by carboxylic acid moiety **14a-c** dramatically diminished *h*CA I inhibition constants to high micromolar values (**14a-c**; K_I_s = 68.09, 82.75 and 11.82 µM, respectively). Furthermore, it was noted that the inclusion of secondary sulfamoyl functionality totally abolished *h*CA I inhibitory effect (**16a-b**; K_I_s > 100 µM) compared to the primary sulfamoyl-appended sulphonamides **8a-c**.

Interestingly, the *in vitro* kinetic data towards the physiologically relevant *h*CA II isoform presented inhibition pattern similar to *h*CA I, Table 1. In a similar fashion, the benzothiazole-derived analogues **8a-c** exhibited moderate to high *h*CA II inhibitory potential with K_I_s spanning between 54.1 and 785.2 nM. While the incorporation of *ortho* or *meta* sulphonamide demonstrated moderate *h*CA II inhibition (**8a-b**; K_I_ = 785.2 and 652.7 nM, respectively), the shifting of this sulfamoyl to *para* position interestingly improved *h*CA II inhibition constant to two-digits value (**8c**; K_I_ = 54.1 nM). To explore the impact of linker length, the obtained results revealed that elongation of the ureido linker in **8c** (K_I_ = 54.1 nm) by one carbon significantly decreased the inhibitory action against *h*CA II by 4-fold (**10**; K_I_ = 204.3 nM), whereas the elongation of this linker by two carbons was more advantageous furnishing the most powerful *h*CA II inhibitor within this series (K_I_ = 28.5 nM) in a similar way to the *h*CA I inhibition profile. Similarly, the appending of carboxyl group as a zinc anchoring moiety **14a-c** in place of the sulfamoyl functionality **8a-c** (K_I_s range 54.1– 785.2 nM) drastically declined *h*CA II inhibition to micromolar level (**14a-c**; K_I_s equal 75.94, 94.13 and 9.47 µM, respectively). In addition, the applying of secondary sulfamoyl group **16a-b** completely abolished *h*CA II inhibitory power similar to *h*CA I inhibition data.

Concerning the inhibitory influence of the here evaluated benzothiazole-based SLC-0111 analogues towards cancer-associated *h*CA IX isozyme (Table 1), the primary sulfamoyl-bearing derivatives **8a-c**, **10** and **12** exerted the most efficient potencies within this study against such enzyme displaying two-digits nanomolar K_I_s spanning a range between 16.4 and 65.3 nM. It is worth to mention that switching of the sulphonamide from *ortho*- and *meta* positions **8a-b** (K_I_s equal 65.3 and 48.9 nM, respectively) to *para* position **8c** enhanced the *h*CA IX inhibitory power (K_I_ equals 31.5 nM), while the elongation of ureido linker in **8c** by one carbon decreased the inhibition potency by the half (**10**; K_I_ = 58.8 nM). On the other hand, extending such a linker by two carbons resulted in the production of the most efficient hCA IX inhibitor within the scope of the present study (**12**; K_I_ = 16.4 nM). Additionally, the replacement of the sulfamoyl zinc binding group in **8a-c** (K_I_ range 31.5 to 65.3 nM) by carboxylic acid functionality sharply lowered the *h*CA IX inhibition constants (**14a-c**; K_I_ = 44.62, 16.28 and 2.41 µM, respectively). Like the *h*CA I and II inhibition outcomes, the incorporation of 3,5-dimethyl-1,2-oxazole-bearing secondary sulphonamide entirely revoked the *h*CA IX inhibitory efficiency (**16b**; K_I_ > 100 µM), whereas the inclusion of thiazole-appended secondary sulphonamide **16a** resulted in very weak *h*CA IX inhibition (K_I_ of 56.09 µM).

In the context of inhibitory activities towards the second tumour-related *h*CA XII isoform, the herein assessed benzothiazole-derived SLC-0111 analogues demonstrated diverse potencies in a similar behaviour as *h*CA I, II and IX isoforms as depicted in Table 1. The primary sulfamoyl-bearing analogues **8a-c**, **10** and **12** showed the most favourable inhibition profile against *h*CA XII isoform demonstrating inhibition constants K_I_s ranged from 29.3 to 57.5 nM. For the ureido linker-grafted sulphonamides **8a-c**, the *para* regioisomer **8c** was the most potent *h*CA XII inhibitor with K_I_ = 29.3 nM, similarly the *ortho* and *meta* regioisomers exhibited potential *h*CA XII inhibition (**8a-b**; K_I_s equal 41.2 and 57.5 nM, respectively). Moreover, the elongation of the ureido linker in **8c** (K_I_ = 29.3 nM) by one or two carbons slightly reduced the inhibition constants (**10** and **12**; K_I_s of 51.2 and 34.7 nM, respectively). As obtained from *h*CA I, II and IX inhibitory investigations, the bioisosteric replacement of the sulphonamide functionality in **8a-c** (K_I_s range 29.3 − 57.5 nM) with carboxylic acid group dramatically decreased the *h*CA XII inhibitory effect (**14a**; K_I_ = 39.86 µM, **14b**; K_I_ = 9.14 µM and **14c**; K_I_ = 8.54 µM). Furthermore, it was noted that the inclusion of secondary sulfamoyl functionality **16a-b** markedly declined *h*CA XII inhibition data, while the grafting of thiazole-bearing sulfamoyl **16a** resulted in K_I_ = 32.5 µM, the introduction of 3,5-dimethyl-1,2-oxazole-bearing secondary sulphonamide **16b** completely abolished the *h*CA XII inhibition (K_I_ > 100 µM).

Collectively, the elicited SAR hinted out that the replacement of 4-fluorophenyl tail in SLC-0111 with benzothiazole motif while maintaining the *para* primary sulfamoyl functionality in conjunction with the elongation of its ureido linker by two carbons furnished the most potent inhibitors towards the tumour-related *h*CA IX with inhibition constants better than the lead SLC-0111 (**8c**, K_I_ = 31.5 nM; **12**, K_I_ = 16.4 nM *vs* SLC-0111, K_I_ = 45 nM). Undesirably such improvement in *h*CA IX inhibition for the promising candidates (**8c** and **12**) was concomitant with the enhancement in inhibition of the ubiquitous *h*CA I (IX/I *S.I*. = 11.5, 3.75, respectively) and II isoforms (IX/II *S.I*. = 1.7 for both) compared to SLC-0111 (IX/I *S.I*. = 112.9; IX/II *S.I*. = 21.3). Consequently, the resulting potent benzothiazole-tethered SLC-0111 analogues **8c** and **12** can be employed as leads for further optimisation to develop promising candidates with superior potency and selectivity towards the cancer-associated isozymes over the physiologically dominant *h*CA I and II isoforms.

#### Antitumor activity towards NCI-60 cancer cell lines

All herein developed benzothiazole-derived sulphonamides **8a-c**, **10**, **12**, **16a-b** and the carboxylic acids **14a-c** were explored for their potential antitumor activities at the National Cancer Institute (NCI-USA) within the Developmental Therapeutic Program, utilising the US-NCI protocol and the sulforhodamine B (SRB) colorimetric assay for cell growth and viability evaluation^32^^,^^37^^,^^42^^,^^43^.

**Figure 3.**
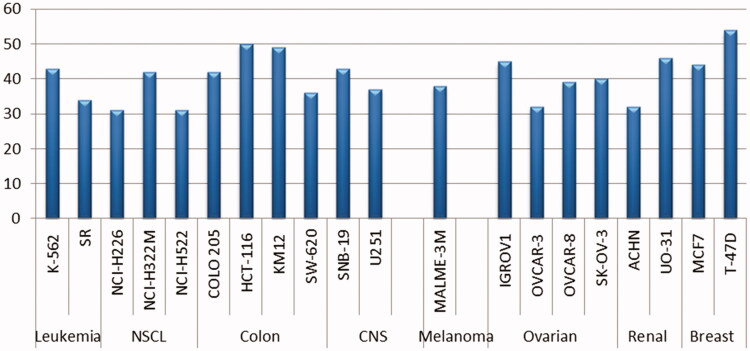
The cancer cell lines that are most vulnerable to the effects of sulphonamide **8b**.

The obtained results revealed that the examined molecules have weak or non-significant antitumor activities towards most NCI cancer cell lines, except **8b.** Sulphonamide **8b** displayed selective anti-proliferative activity (GI > 30%) towards twenty cancer cell lines belonging to all tumour subpanels, except the prostate cancer subpanel, with GI% range of 31–54% (Figure 3). The best growth inhibitory activity of **8b** (GI = 54%) was observed for the breast T-47D cancer cell line. Figure 3 provides a summary of the cancer cell lines that are most vulnerable to the effects of benzothiazole-derived sulphonamide **8b**.

Sulphonamide **8c** exerted cell growth inhibition (GI) equals about 20% towards non-small cell lung cancer (EKVX) and breast cancer (MCF7 and T-47D) cell lines, as well as GI = 24% towards renal cancer (UO-31) cell line. Sulphonamide **10** displayed GI equals about 20% against non-small cell lung cancer (NCI-H226 and EKVX), and breast cancer (MCF7 and T-47D) cell lines, in addition, it exerted about 25% GI towards ovarian cancer (IGROV1) and CNS cancer (SNB-75) cell lines. Superiorly, the renal cancer (UO-31) cell line was the most sensitive one to the effect of sulphonamide **10** with a GI value of 33%. Moreover, non-small cell lung cancer (EKVX), ovarian cancer (IGROV1) and CNS cancer (SNB-75), and renal cancer (UO-31) and breast cancer (MCF7 and T-47D) cell lines were the most susceptible cells to the impact of sulphonamides **16a** and **16b** with GI % = 27, 28, 29, 37, 24, 22 for **16a**, and GI % = 19, 24, 37, 29, 24, 16 for **16b**.

Regarding the anticancer activities of series **14**, carboxylic acid derivative **14a** was found to possess a moderate growth inhibitory effect against non-small cell lung cancer (EKVX), ovarian cancer (IGROV1), CNS cancer (SNB-75), and renal cancer (CAKI-1 and UO-31) cell lines with inhibition % 22, 27, 20, 20, and 32, respectively. Also, carboxylic acid derivative **14b** displayed GI more than 20% against ovarian cancer (IGROV1), CNS cancer (SNB-75), and renal cancer (UO-31) cell lines, whereas compound **14c** exerted GI more than 20% against ovarian cancer (IGROV1), non-small cell lung cancer (EKVX), renal cancer (UO-31) and breast cancer (MCF7 and T-47D) cell lines.

#### Anti-proliferative activity towards breast cancer cell lines

The inhibition constants presented in Table 1 highlighted that sulphonamide **8b** elicited an excellent selectivity towards *h*CA IX and XII over the off-target *h*CA I, with selective indexes (SIs) equal 82.6 and 70.2, respectively. In addition, only sulphonamide **8b** demonstrated excellent selectivity towards hCA IX and XII in comparison to hCA II, with SI values of 13.3 and 11.3, respectively. As a consequence of this, sulphonamide **8b** maintained both its activity and its selectivity with regard to the *h*CA IX and XII isoforms that are associated with cancer. In addition, according to the findings of the US-NCI assay that was discussed before, sulphonamide **8b** was determined to be the most effective anticancer molecules herein reported. As a consequence, sulphonamide **8b** was evaluated for its anticancer effect towards breast cancer (T-47D and MCF-7) cell lines within the hypoxic conditions, using the SRB assay. The assay results that presented in Table 2 showed that sulphonamide **8b** had good IC_50_ values (6.73 ± 0.28 and 9.16 ± 0.70) against T-47D and MCF-7 cells, respectively.

**Table 2.** Anticancer activity of target benzothiazole-based sulphonamide **8b** towards breast T-47D and MCF-7 cancer cell lines under hypoxic conditions.

Com.	IC_50_ (µM)	
	T-47D	MCF-7
**8b**	6.73 ± 0.28	9.16 ± 0.70
**Doxorubicin**	7.31 ± 0.49	6.52 ± 0.38

## Conclusions

This study developed different series of benzothiazole-based sulphonamides **8a-c, 10, 12, 16a-b** and carboxylic acids as novel SLC-0111 analogues, and assessed their CA inhibitory effects towards CA I, II, IX and XII isoforms. Different drug design approaches were utilised. The benzothiazole motif was appended to the ureido linker instead of the SLC-0111 fluorophenyl tail to produce compounds **8c** and its regioisomers **8a-b**. In addition, the ureido spacer was elongated by methylene or ethylene groups to afford the counterparts **10** and **12**. In turn, the functionality of the primary ZBG was either replaced by carboxylic acid or it was substituted in order to produce the carboxylic acid derivatives **14a-c** and the secondary sulphonamide-based SLC-0111 analogues **16a-b**, respectively. The elicited SAR, from this study, hinted out that the introduction of *para* primary sulfamoyl functionality along with elongation of the ureido linker by two carbons were more beneficial for the cancer-related *h*CA IX and XII inhibition. The primary sulfamoyl-bearing analogues **8a-c**, **10** and **12** disclosed the most favourable inhibition profile against tumour-related *h*CA IX and XII isoforms demonstrating inhibition constants ranged from 16.4 to 65.3 nM and from 29.3 to 57.5 nM, respectively. Moreover, all herein developed benzothiazole-derived sulphonamides **8a-c**, **10**, **12**, **16a-b** and the carboxylic acids **14a-c** were explored for their potential antitumor activities at the NCI-National Cancer Institute. The examined molecules have weak or non-significant antitumor activities towards most NCI cancer cell lines, except sulphonamide **8b** which displayed selective anti-proliferative activity (GI > 30%) towards twenty cancer cell lines belonging to all tumour subpanels, except the prostate cancer subpanel. It is interesting to mention that **8b** elicited an excellent selectivity towards *h*CA IX and XII over the off-target hCA I, with selective indexes equal to 82.6 and 70.2, respectively. Additionally, it demonstrated good selectivity towards hCA IX and XII over hCA II with selective indexes of 13.3 and 11.3, respectively. Furthermore, the cytotoxicity SRB assay showed that sulphonamide **8b** had good IC_50_ values (6.73 ± 0.28 and 9.16 ± 0.70) against breast cancer T-47D and MCF-7 cells, respectively.

## Supplementary Material

Supplemental Material
