# Supplementary material for: Design and synthesis of benzothiazole-based SLC-0111 analogues as new inhibitors for the cancer-associated carbonic anhydrase isoforms IX and XII
Source: J Enzyme Inhib Med Chem. 2022 Sep 22;37(1):2635–43. doi: 10.1080/14756366.2022.2124409 (PMC9518259; doi:10.1080/14756366.2022.2124409)
Supplement: Supplemental Material [file IENZ_A_2124409_SM6014.pdf]

Supporting Information

Design and synthesis of benzothiazole-based SLC-0111 analogues as new inhibitors for the cancer-associated carbonic anhydrase isoforms IX and XII

Tarfah Al-Warhi, Mostafa M. Elbadawi, Alessandro Bonardi, Alessio Nocentini, Ahmed A. Al-Karmalawy, Nada Aljaeed, Ohoud J. Alotaibi, Hatem A. Abdel-Aziz, Claudiu T. Supuran, Wagdy M. Eldehna

* Corresponding authors. E-mail addresses: wagdy2000@gmail.com (W.M. Eldehna), claudiu.supuran@unifi.it (C.T. Supuran).

Tables of Contents

1. Physical and spectral data for target benzothiazole derivatives 2-4

2. Carbonic Anhydrase Inhibition Assay 5

1. Physical and spectral data for target benzothiazole derivatives

2-(3-(Benzo[d]thiazol-2-yl)ureido)benzenesulfonamide (8a)

White crystals (yield 68%), m.p. 227-229°C; IR (KBr, ν cm^{-1}), 3259, 3180 (NH_2, NH), 1665 (C=O), 1267, 1166 (SO_2); ^{1}HNMR (DMSO-d_6) *δ ppm*: 9.99 (s, 1H, NH), 8.26 (s, 1H, NH), 7.86 (d, 1H, Ar-H, *J* = 7.6 Hz), 7.67 (d, 2H, Ar-H, *J* = 8.4 Hz), 7.61 (t, 1H, Ar-H, *J* = 8.0 Hz), 7.41 (t, 2H, Ar-H, *J* = 8.0 Hz), 7.36 (d, 2H, Ar-H, *J* = 8.4 Hz), 7.12 (s, 2H, NH_2, -SO_2NH_2); Elemental Analysis for $C_{14}H_{12}N_4O_3S_2$: Calcd C, 48.27; H, 3.47; N, 16.08, Found C, 48.09; H, 3.49; N, 16.15.

3-(3-(Benzo[d]thiazol-2-yl)ureido)benzenesulfonamide (8b)

White crystals (yield 70%), m.p. 236-239°C; IR (KBr, ν cm^{-1}), 3360, 3274 (NH_2, NH), 1680 (C=O), 1322, 1151 (SO_2); ^{1}HNMR (DMSO-d_6) *δ ppm*: 9.53 (s, 1H, NH); 8.19 (s, 1H, NH), 7.90 (d, 1H, Ar-H, *J* = 7.6 Hz), 7.16-7.67 (m, 9H, Ar-H); ^{13}CNMR (DMSO-d_6) *δ ppm*: 116.17, 117.05, 120.38, 122.11, 122.22, 123.49, 126.58, 128.58, 128.66, 129.36, 130.04, 139.65, 145.29, 149.55; Elemental Analysis for $C_{14}H_{12}N_4O_3S_2$: Calcd C, 48.27; H, 3.47; N, 16.08, Found C, 48.13; H, 3.46; N, 16.16.

4-(3-(Benzo[d]thiazol-2-yl)ureido)benzenesulfonamide (8c)

White crystals (yield 73%), m.p. 254-256°C; IR (KBr, ν cm^{-1}), 3365, 3271 (NH_2, NH), 1666 (C=O), 1323, 1151 (SO_2); ^{1}HNMR (DMSO-d_6) *δ ppm*: 9.60 (s, 1H, NH); 9.13 (s, 1H, NH), 7.97 (d, 1H, Ar-H, *J* = 8.0 Hz), 7.77 (d, 2H, Ar-H, *J* = 8.4 Hz), 7.69-7.63 (m, 3H, Ar-H), 7.23-7.34 (m, 4H, 2 Ar-H and 2H of -SO_2NH_2); Elemental Analysis for $C_{14}H_{12}N_4O_3S_2$: Calcd C, 48.27; H, 3.47; N, 16.08, Found C, 48.11; H, 3.48; N, 16.12.

4-((3-(Benzo[d]thiazol-2-yl)ureido)methyl)benzenesulfonamide (10)

White crystals (yield 75%), m.p. 243-245°C; IR (KBr, ν cm^{-1}), 3236, 3327 (NH_2, NH), 1677 (C=O), 1300, 1151 (SO_2); ^{1}HNMR (DMSO-d_6) *δ ppm*: 9.51 (s, 1H, NH), 8.39 (d, 1H, Ar-H, *J* = 7.6 Hz), 8.07 (d, 1H, Ar-H, *J* = 8.0 Hz), 7.83 (d, 2H, Ar-H, *J* = 8.4 Hz), 7.78-7.69 (m, 3H, 2 Ar-H and 1 NH), 7.52 (d, 2H, Ar-H, *J* = 8.4 Hz), 7.25 (s, 2H, NH_2, -SO_2NH_2), 4.13 (d, 2H,

Information Classification: General

C*H*$_2$- NH, *J* = 6.8 Hz); Elemental Analysis for $C_{15}H_{14}N_4O_3S_2$: Calcd C, 49.71; H, 3.89; N, 15.46, Found C, 49.89; H, 3.91; N, 15.38.

4-(2-(3-(Benzo[d]thiazol-2-yl)ureido)ethyl)benzenesulfonamide (12)

White crystals (yield 83%), m.p. 261-263°C; IR (KBr, ν cm^{-1}), 3287, 3340 (NH_2, NH), 1669 (C=O), 1314, 1153 (SO_2); ^{1}HNMR (DMSO-d_6) *δ ppm*: 9.30 (s, 1H, NH), 8.22 (d, 1H, Ar-H, *J* = 7.6 Hz), 8.13 (d, 1H, Ar-H, *J* = 8.0 Hz), 7.76 (d, 2H, Ar-H, *J* = 8.0 Hz), 7.66-7.58 (m, 3H, 2Ar-H and 1 NH), 7.45 (d, 2H, Ar-H, *J* = 8.0 Hz), 7.29 (s, 2H, NH_2, -SO_2NH_2), 3.58 (q, 2H, CH_2-C*H*$_2$-NH, *J* = 6.4 Hz), 2.99 (t, 2H, C*H*$_2$-CH_2-NH, *J* = 6.8 Hz); Elemental Analysis for $C_{16}H_{16}N_4O_3S_2$: Calcd C, 51.05; H, 4.28; N, 14.88, Found C, 50.87; H, 4.31; N, 14.96.

2-(3-(Benzo[d]thiazol-2-yl)ureido)benzoic acid (14a)

White crystals (yield 61%), m.p. 271-273°C; IR (KBr, ν cm^{-1}), 3192 (OH), 3095 (NH), 1686 (C=O); ^{1}HNMR (DMSO-d_6) *δ ppm*: 11.20 (s, 1H, COOH), 9.53 (s, 1H, NH), 8.19 (s, 1H, NH), 7.04-7.53 (m, 8H, Ar-H); Elemental Analysis for $C_{15}H_{11}N_3O_3S$: Calcd C, 57.50; H, 3.54; N, 13.41, Found C, 57.64; H, 3.55; N, 13.37.

3-(3-(Benzo[d]thiazol-2-yl)ureido)benzoic acid (14b)

White crystals (yield 80%), m.p. 262-263°C; IR (KBr, ν cm^{-1}), 3286 (OH), 3075 (NH), 1663 (C=O); ^{1}HNMR (DMSO-d_6) *δ ppm*: 9.17 (s, 1H, NH), 8.81 (s, 1H, Ar-H), 8.16 (s, 1H, NH), 7.69 (d, 1H, Ar-H, *J* = 8.4 Hz), 7.53-7.59 (m, 2H, Ar-H), 7.40-7.47 (m, 2H, Ar-H), 7.23-7.27 (m, 2H, Ar-H); ^{13}CNMR (DMSO-d_6) *δ ppm*: 167.74, 153.17, 151.52, 144.54, 140.21, 131.67, 130.21, 128.42, 124.26, 123.73, 122.85, 119.93, 110.11, 106.21; Elemental Analysis for $C_{15}H_{11}N_3O_3S$: Calcd C, 57.50; H, 3.54; N, 13.41, Found C, 57.63; H, 3.52; N, 13.36.

4-(3-(Benzo[d]thiazol-2-yl)ureido)benzoic acid (14c)

White crystals (yield 89%), m.p. 285-287°C; IR (KBr, ν cm^{-1}), 3276 (OH), 3078 (NH), 1679 (C=O); ^{1}HNMR (DMSO-d_6) *δ ppm*: 12.18 (s, 1H, COOH), 9.55 (s, 1H, NH), 8.23 (s, 1H, NH), 7.65-7.90 (m, 5H, Ar-H), 7.26-7.46 (m, 3H, Ar-H); Elemental Analysis for $C_{15}H_{11}N_3O_3S$: Calcd C, 57.50; H, 3.54; N, 13.41, Found C, 57.61; H, 3.53; N, 13.47.

Information Classification: General

3-(3-(Benzo[d]thiazol-2-yl)ureido)-N-(thiazol-2-yl)benzenesulfonamide (16a)

White crystals (yield 78%), m.p. 214-216°C; IR (KBr, ν cm^{-1}), 3282, 3356 (NH), 1713 (C=O), 1292, 1144 (SO_2); ^{1}HNMR (DMSO-d_6) *δ ppm*: 12.56 (s, 1H, NH), 9.35 (s, 1H, NH), 8.80 (s, 1H, NH), 7.73 (d, 2H, Ar-H, *J* = 8.0 Hz), 7.64 (s, 2H, Ar-H), 7.53 (d, 1H, Ar-H, *J* = 7.6 Hz), 7.45 (d, 1H, Ar-H, *J* = 7.2 Hz), 7.18-7.30 (m, 3H, Ar-H), 6.80 (d, 1H, Ar-H, *J* = 4.8 Hz); Elemental Analysis for $C_{17}H_{13}N_5O_3S_3$: Calcd C, 47.32; H, 3.04; N, 16.23, Found C, 47.17; H, 3.07; N, 16.26.

3-(3-(Benzo[d]thiazol-2-yl)ureido)-N-(3,4-dimethylisoxazol-5-yl)benzenesulfonamide (16b)

White crystals (yield 72%), m.p. 229-231°C; IR (KBr, ν cm^{-1}), 3244, 3307 (NH), 1723 (C=O), 1323, 1159 (SO_2); ^{1}HNMR (DMSO-d_6) *δ ppm*: 10.03 (s, 1H, NH), 8.24 (s, 1H, NH), 8.13 (s, 1H, NH), 7.75 (d, 1H, Ar-H, *J* = 7.6 Hz), 7.67 (d, 2H, Ar-H, *J* = 8.4 Hz), 7.12-7.41 (m, 5H, Ar-H), 2.27 (s, 3H, CH_3), 1.90 (s, 3H, CH_3); Elemental Analysis for $C_{19}H_{17}N_5O_4S_2$: Calcd C, 51.46; H, 3.86; N, 15.79, Found C, 51.67; H, 3.88; N, 15.73.

2. Carbonic anhydrase inhibition assay

The carbonic anhydrase catalyzed CO_2 hydration actions for the benzothiazole derivatives (**8a-c, 10, 12, 14a-c** and **16a-b**) were assayed utilizing an instrument of Applied Photophysics stopped-flow [3], as described previously. The enzymes are recombinant proteins prepared in our lab. Phenol red (at a concentration of 0.2 mM) has been used as indicator, working at the absorbance maximum of 557 nm, with 20 mM Hepes (pH 7.5) as buffer, and 20 mM Na_2SO_4 (for maintaining constant the ionic strength), following the initial rates of the CA-catalyzed CO_2 hydration reaction for a period of 10-100 s. The CO_2 concentrations ranged from 1.7 to 17 mM for the determination of the kinetic parameters and inhibition constants. For each inhibitor at least six traces of the initial 5-10% of the reaction have been used for determining the initial velocity. The uncatalyzed rates were determined in the same manner and subtracted from the total observed rates. Stock solutions of inhibitor (0.1 mM) were prepared in distilled-deionized water and dilutions up to 0.01 nM were done thereafter with the assay buffer. Inhibitor and enzyme solutions were preincubated together for 15 min at room temperature prior to assay, in order to allow for the formation of the E-I complex. The inhibition constants were obtained by non-linear least-squares methods using PRISM 3 and the Cheng-Prusoff equation, and represent the mean from at least three different determinations.
